# Spectroscopic, electrochemical, and kinetic trends in Fe(III)–thiolate disproportionation near physiologic pH

**DOI:** 10.1007/s00775-024-02051-3

**Published:** 2024-05-09

**Authors:** Levi A. Ekanger, Ruhi K. Shah, Matthew E. Porowski, Zach Ziolkowski, Alana Calello

**Affiliations:** Department of Chemistry, The College of New Jersey, Ewing, NJ 08628 USA

**Keywords:** Electrochemistry, Electron paramagnetic resonance, Ligand binding, Kinetics, Redox

## Abstract

In addition to its primary oxygen-atom-transfer function, cysteamine dioxygenase (ADO) exhibits a relatively understudied anaerobic disproportionation reaction (ADO-Fe(III)-SR → ADO-Fe(II) + ½ RSSR) with its native substrates. Inspired by ADO disproportionation reactivity, we employ [Fe(tacn)Cl_3_] (tacn = 1,4,7-triazacyclononane) as a precursor for generating Fe(III)–thiolate model complexes in buffered aqueous media. A series of Fe(III)–thiolate model complexes are generated in situ using aqueous [Fe(tacn)Cl_3_] and thiol-containing ligands cysteamine, penicillamine, mercaptopropionate, cysteine, cysteine methyl ester, *N*-acetylcysteine, and *N*-acetylcysteine methyl ester. We observe trends in UV–Vis and electron paramagnetic resonance (EPR) spectra, disproportionation rate constants, and cathodic peak potentials as a function of thiol ligand. These trends will be useful in rationalizing substrate-dependent Fe(III)–thiolate disproportionation reactions in metalloenzymes.

**Graphical abstract:**

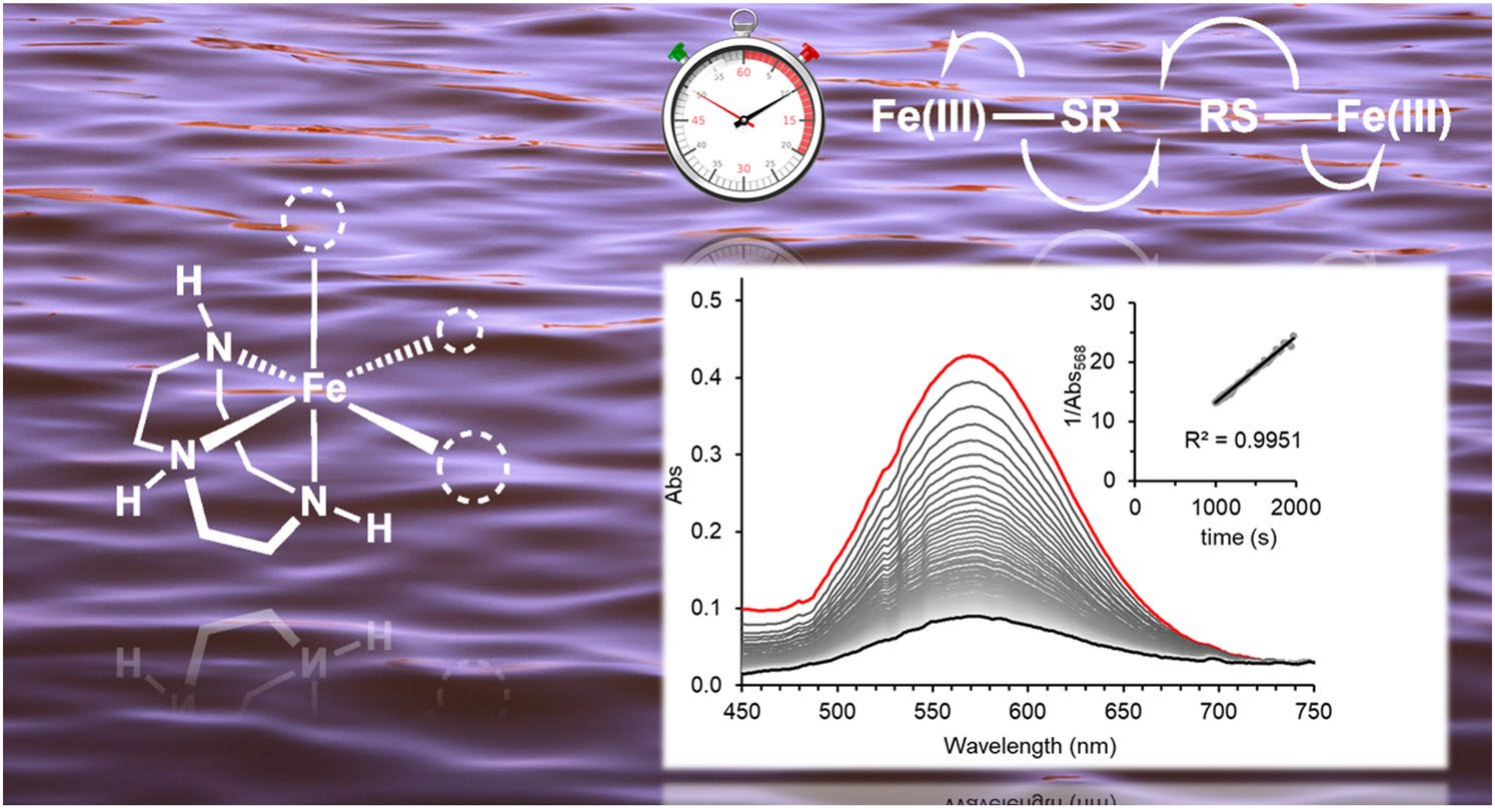

**Supplementary Information:**

The online version contains supplementary material available at 10.1007/s00775-024-02051-3.

## Introduction

**Fig. 1:**
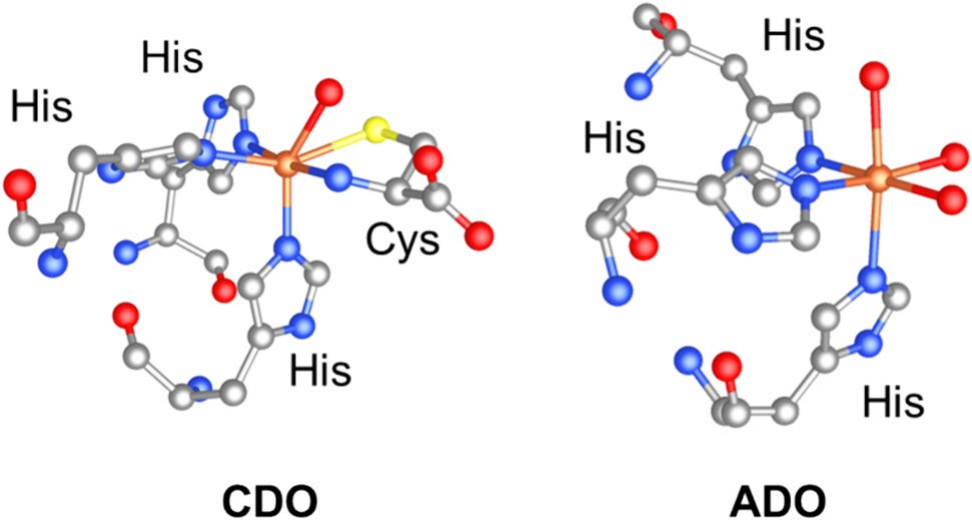
Simplified coordination environments at CDO (left) and ADO (right) active sites demonstrating the 3N binding mode of histidine (His). Cysteine (Cys) is bound to CDO in a bidentate mode while ADO contains no substrate. Protein crystal structures PDB: 4Z82 and PDB: 7LVZ were used for CDO and ADO, respectively, where carbon is gray, nitrogen is blue, oxygen is red, and iron is orange

Mononuclear non-heme Fe enzymes are diverse and ubiquitous with functionality broadly divided into oxygen or substrate activation [1–3]. Thiol dioxygenases, such as cysteine dioxygenase (CDO) and cysteamine dioxygenase (ADO), transform biologic thiols into sulfinates and are the largest family of non-heme Fe(II)-dependent enzymes in which Fe is bound to the protein by a histidine triad [1, 4–11]. Given the ubiquity and importance in nature of thiol dioxygenases, including CDO, ADO, and plant cysteine dioxygenase (PCO), the Pfam-defined CDO_I family and PCO_ADO family have received increasing attention to better understand their structures and functions [1, 3, 6, 12–15]. Thiol dioxygenase active sites feature a mononuclear, non-heme Fe center bound by three histidine residues forming a facial 3N binding mode (Fig. 1). The remaining three coordination sites on Fe are either occupied by three water molecules in the absence of substrate or by a combination of thiol substrate, water, or dioxygen. It should be noted that the CDO_I and PCO_ADO families are functionally quite dissimilar and share low sequence homology beyond the cupin fold three-histidine triad contributing to the first coordination sphere of the Fe center [1]. Within the CDO active-site cysteine binds to Fe through thiolate S and amine N donor atoms (Fig. 1) [16, 17]. Bidentate substrate binding is also observed at the mercaptopropionate dioxygenase (MDO) active site [14]. In contrast, evidence strongly suggests monodentate substrate binding at the ADO active site [1, 7, 8, 18]. When compared to CDO and MDO, ADO is unique in its substrate binding mode and scope.

In addition to catalyzing thiolate-to-sulfinate transformations, ADO exhibits disproportionation chemistry. Disproportionation is a type of reduction–oxidation reaction in which species with the same oxidation state combine to yield one of higher oxidation state and one of lower oxidation state [19]. In the case of Fe(III)–thiolate disproportionation, the Fe(III) center is reduced to Fe(II) with concomitant oxidation of thiolate yielding the corresponding disulfide (Fe(III)-SR → Fe(II) + ½ RSSR). Disproportionation reactivity is speculated to have a role in ADO function as a mechanism to reduce Fe(III) to Fe(II). Considering Fe(II) is required to activate O_2_ for dioxygenase activity, a mechanism to restore Fe(II) via native thiol substrates might be advantageous to maintain the Fe(II) resting state. Disproportionation reactions are documented between ADO and both cysteamine and the N-terminus of regulator of G signaling 5 (RGS5) [18], but this reactivity remains relatively understudied. In addition, it is unclear why disproportionation chemistry is observed between ADO-Fe(III) and its native substrates while CDO-Fe(III) forms stable complexes with cysteine. To provide additional insight into the disproportionation of Fe(III)–thiolate intermediates, we sought to investigate the effect of thiol ligands on Fe(III)–thiolate disproportionation rates and cathodic peak potentials.

To investigate how thiol ligands affect disproportionation rates, we elected to pursue model chemistry using an Fe complex. Given the ubiquity of Fe and S in biologic systems, Fe(III)–thiolate chemistry has been studied in numerous contexts [20–26]. Early model chemistry studies focused on Fe(III)–cysteine and related Fe(III)–thiolate interactions that form colorful solutions from S → Fe(III) charge transfer transitions. It is well documented that Fe(III)–thiolate model complexes decay to colorless products under aerobic or anaerobic conditions consistent with a disproportionation process. Transient formation of colorful model complexes enables colorimetric analyses to quantify their rates of formation and disappearance. For example, the kinetics of Fe(III)–cysteine and –thiolate interactions have been studied using simple (i.e., non-chelated) Fe(III) salts in acidic or alkaline media [27–30]. A consistent observation among prior studies is the decay of Fe(III)–thiolate model complexes following second-order kinetics consistent with a bimolecular disproportionation process (2 Fe(III)-SR → 2 Fe(II) + RSSR). Moreover, both early and modern studies use cysteine analogs, such as cysteamine and mercaptopropionate, to study the effect of ligand binding on electronic structure and reactivity [30, 31].

Prior studies on the anaerobic oxidation of cysteine by Fe(III) have experimental limitations that do not fit within the context of ADO disproportionation chemistry. First, the use of non-chelated Fe(III) ions in prior studies do not replicate the 3N facial coordination environment of thiol dioxygenases. Second, the use of non-chelated Fe(III) ions necessitates the use of acidic or alkaline reaction solutions to prevent Fe(III)–hydroxide olation and precipitation near neutral pH values. The use of either highly acidic or alkaline pH values are not ideal considering substrate binding occurs in a physiologically relevant pH range in thiol dioxygenases. For example, CDO binds cysteine optimally at pH ~ 7.4 [6]. Accordingly, the use of chelated Fe(III) is required to provide 3N facial coordination and to enable studies in solutions buffered in a physiologically relevant pH range.

Motivated by early studies of Fe(III)–cysteine reaction kinetics, we turned to contemporary studies focused on thiol dioxygenase model chemistry to identify a suitable ligand for our studies. Thiol dioxygenase active sites are commonly modeled using tridentate ligands with nitrogen donor atoms to replicate the 3N facial coordination environment found in thiol dioxygenases [32–40]. We elected to use the ligand 1,4,7-triazacyclononane (tacn) because it is a standard tridentate ligand platform used to study modern thiol dioxygenase model chemistry. For example, *N*-methyl and -isopropyl tacn derivatives were used to synthesize and characterize thiol dioxygenase model complexes capable of catalyzing S-oxygenation and disproportionation chemistry [32]. We emphasize here that the intention of this study is not to replicate secondary coordination sphere effects that give rise to dissimilar function especially between the CDO_I and PCO_ADO thiol dioxygenase families. Instead, our aim is to study fundamental characteristics of aqueous Fe(III)–thiolate disproportionation near physiologic pH which might have some relevance to anaerobic thiol dioxygenase disproportionation.

**Fig. 2:**
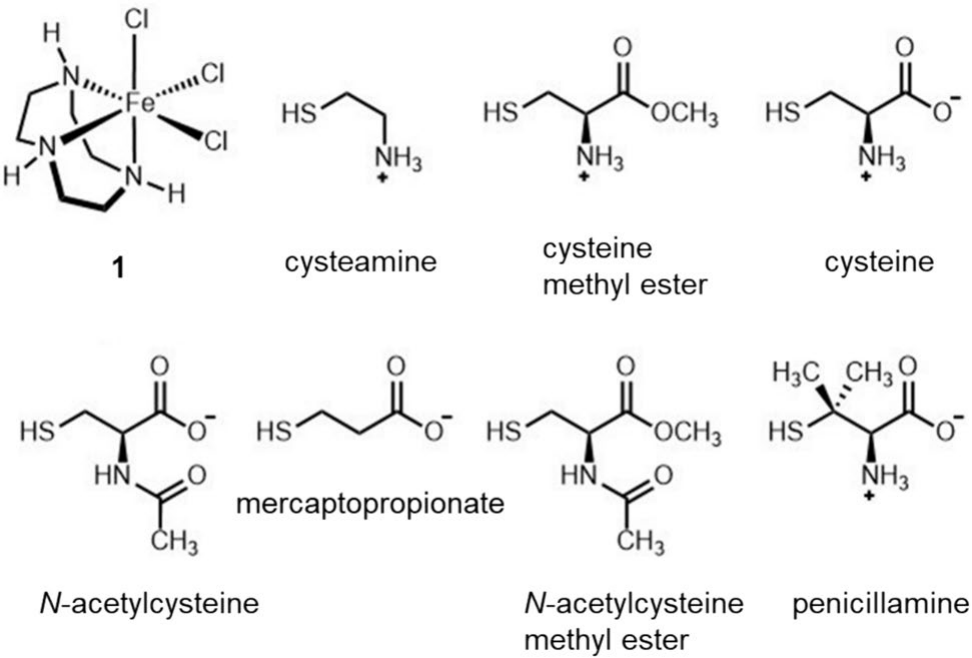
Structures of [Fe(tacn)Cl_3_] (**1)** and thiol ligands used in this study. The top row features **1**, cysteamine, cysteine methyl ester, and cysteine. The bottom row features *N*-acetylcysteine, mercaptopropionate, *N*-acetylcysteine methyl ester, and penicillamine

Here we report spectroscopic, electrochemical, and kinetic trends in a series of Fe(III)–thiolate model complexes generated in situ within 37 °C buffered solution. Model complex formation is initiated by combining aqueous [Fe(tacn)Cl_3_] (**1(aq)**) with cysteamine or cysteamine analogs including penicillamine, mercaptopropionate, cysteine, cysteine methyl ester, *N*-acetylcysteine, and *N*-acetylcysteine methyl ester (Fig. 2). We also report the synthesis and characterization of the most stable model complex containing Fe(III)-bound penicillamine to better understand its coordination environment and electronic structure using FT-IR, UV–Vis, EPR, and NMR spectroscopies and magnetic susceptibility measurements.

## Experimental section

### General methods

All reagents were ACS reagent grade or better and used as received unless otherwise noted. Deionized water was purified to a resistivity of 18 Mohm cm and used to prepare all solutions.

### Synthesis of [Fe(tacn)Cl_3_] (1)

Following a reported procedure [41], separate ethanolic solutions of 1,4,7-triazacyclononane (0.485 g, 3.75 mmol) and FeCl_3_·(H_2_O)_6_ (1.160 g, 4.29 mmol) were combined to yield a yellow precipitate. The precipitate was isolated by vacuum filtration with a cold ethanol rinse and air dried to yield 1.029 g (94% yield) of **1** as a fine, yellow powder. Aqueous solutions of **1** are denoted as **1(aq)**.

### Synthesis of [Fe(tacn)(pen)]BPh_4_ (2)

l-Penicillamine (pen, 0.700 g, 4.69 mmol) and NaOH (0.295 g, 7.38 mmol) were combined and stirred in water (35 mL). After all solids dissolved, **1(aq)** (0.100 L, 9.19 mM, 0.919 mmol) was added giving rise to a deep purple solution. After stirring for 5 min, aqueous NaBPh_4_ (0.150 L, 58.4 mM, 8.77 mmol) was added to the purple solution producing a fine, blue precipitate. The reaction mixture was stirred in an ice bath for 1 h before precipitate was isolated by centrifugation (50 mL conical tubes). Vacuum filtration is time consuming and not advised because the reaction mixture is highly viscous caused by excess NaBPh_4_ in solution. Precipitate was rinsed with cold-water within centrifuge tubes and isolated by centrifugation to yield a blue powder. After drying under reduced pressure overnight, the blue powder was dissolved in a minimal volume of THF and precipitated by the addition of diethyl ether. Isolation by vacuum filtration yielded a dark purple powder. Yield = 0.329 g (55%). Selected IR bands (KBr pellet): ν (cm^–1^) = 3242 (w, (N–H)), 1646 (s, ν(C=O)). ^1^H NMR (400 MHz, CD_3_CN, 17 °C): δ (ppm) 29.1, 19.2, 10.6, [7.28 (2H), 7.00 (2H), 6.85 (1H); tetraphenylborate counterion], 2.26 (H_2_O), 1.94 (solvent residual peak), –3.0, –13.4, –20.9. Magnetic susceptibility balance:* µ*_eff_ = 1.67 *µ*_B_; Evans method (400 MHz, CD_3_CN): *µ*_eff_ = 1.68 *µ*_B_. Anal. Calcd for FeC_35_H_44_N_4_O_2_SB·H_2_O: C, 62.79; H, 6.93; N, 8.37. Found: C, 62.17; H, 7.15; N, 7.99.

### Kinetics measurements

All kinetics measurements were performed under a N_2_ atmosphere (2–4% H_2_, < 1 ppm O_2_) within an anaerobic chamber (Coy Laboratories). Solutions were prepared in degassed water. Reactions were performed in water or *N*,*N*-bis(2-hydroxyethyl)-2-aminoethanesulfonate (BES) buffer (20 mM, pH 7.1 or 7.5). It should be noted that **1(aq)** appears to induce water hydrolysis over the course of approximately 6 h. Over this period **1(aq)** transitions from a clear, yellow to clear, orange solution. To avoid the formation of hydrolysis products, we prepared fresh solutions of **1(aq)** at most 2 h from the start of each kinetics experiment. Kinetics experiments were initiated by heating separate solutions of thiol ligand (10.0 mM) and **1(aq)** (1.00 mM) at 37 °C. While solutions were heating, a quartz cuvette (Starna Cells, Spectrosil Far UV Quartz Cuvette, 9B-Q-10) was heated at 37 °C within a UV–Vis spectrophotometer (DeNOVIX DS-C). After heating for 20 min, 500 μL of each solution was added to the pre-heated quartz cuvette to initiate a reaction. UV–Vis spectra were collected every 5 s for 5 min, every 30 s for 15 min, and every 2 min for 40 min. Second-order rate constants were calculated using absorbance at 568 nm for all reactions except with cysteamine in which absorbance at 618 nm was used. Rate constant calculations were not performed for reactions with cysteine methyl ester or *N*-acetylcysteine methyl ester.

### Electron paramagnetic resonance spectroscopy

Continuous-wave EPR spectra were collected using a Bruker EMXPlus spectrometer with a standard high sensitivity X-band resonator. Samples were prepared by adding **1** (14.6 mg, 0.0501 mmol) to a 5.00 mL solution of thiol ligand (20 mM) in buffer (20 mM BES, 138 mM NaCl, pH 7.5). Samples were prepared within an anaerobic chamber, loaded into thick-walled quartz EPR tubes (4 mm OD), immediately capped and removed from the chamber before flash freezing in liquid nitrogen. All spectra were acquired at 77 K using a quartz liquid nitrogen Dewar flask installed within the resonator.

### Electrochemistry

Cyclic voltammetry experiments were performed using Go Direct screen-printed electrodes (Vernier) featuring a carbon working electrode, carbon counter electrode, and Ag/AgCl reference electrode all of which are screen printed on a single substrate. The screen-printed electrode was used with a Go Direct (Vernier) potentiostat operated via Bluetooth within the same anaerobic chamber used for kinetics experiments (N_2_ atmosphere, 2–4% H_2_, < 1 ppm O_2_). Acquisition parameters were two segments, an initial potential of − 1000 mV, switching potential of 500 mV, final potential of − 1000 mV, and a scan rate of 750 mV/s. Samples were prepared by adding **1** (29.1 mg, 0.100 mmol) to a 10.0 mL solution of thiol ligand (10 mM) in buffer (20 mM BES, 138 mM NaCl, pH 7.5). The resulting solutions were mixed by swirling and the screen-printed electrode, which was rinsed in deionized water between measurements, was immediately placed into solution to record cyclic voltammograms.

### LC–MS

Reactions were prepared in the same manner used for kinetics measurements except **1(aq)** and ligand had final concentrations of 10 and 20 mM, respectively. After 1 h and still under an anaerobic atmosphere, solutions were transferred to a vial using a syringe and syringe-driven filter unit (0.2 micron, hydrophilic). Reaction solutions were analyzed by LC–MS (Agilent Technologies 1260 Infinity HPLC, Poroshell 120 EC-C18 2.7-micron column, and Agilent Technologies 6130 Quadrupole mass spectrometer).

### Physical methods

Elemental analyses were performed at Intertek Pharmaceutical Services in Whitehouse, NJ. Infrared (IR) spectra were obtained with a Perkin-Elmer Spectrum Two FT-IR spectrometer. ^1^H NMR spectra were collected in deuterated solvents using a Bruker Biospin Ascend 400 MHz spectrometer and referenced to residual solvent signal.

### Magnetic susceptibility

The effective magnetic moment of **2** was determined using a magnetic susceptibility balance (Johnson Matthey Mark I) and by the Evans NMR method [42, 43]. Samples were prepared for the Evans method using a 5 mm coaxial insert (Norell) filled with a diamagnetic reference solution of CD_3_CN containing TMS (1.5% v/v). The outer NMR tube contained a solution of **2** (25.0 mM) in CD_3_CN containing TMS (1.5% v/v). The paramagnetic frequency shift of the TMS proton resonance in Hz was used to calculate the effective magnetic moment.

## Results and discussion

### Complex 1 forms reactive model complexes

We selected tacn-chelated Fe(III) to mimic the facial N-donor atoms in thiol dioxygenase active sites and to enable our reactions to occur in aqueous solution near physiologic pH. We first tested if Fe(III)–thiolate model complexes form when starting from **1** in deionized water. Mixing aqueous solutions of **1(aq)** and excess ligand (10 equivalents thiol) produced violet (*λ*_max_ = 568 nm) model complexes with cysteine, penicillamine, *N*-acetylcysteine, and mercaptopropionate while blue (*λ*_max_ = 618 nm) model complexes were produced using cysteine methyl ester and cysteamine. The difference in *λ*_max_ strongly suggests distinct coordination chemistry between the two groups. While the exact binding mode is not known, it is plausible that cysteamine and cysteine methyl ester bind similarly through S and N-donor atoms. A common feature of the ligands that produce violet model complexes is a carboxylate group suggesting a binding interaction involving S and O donor atoms. No colorimetric changes were observed using the negative control *N*-acetylcysteine methyl ester. Violet and blue model complexes decayed to colorless solutions, but color was reestablished upon aeration. The most plausible interpretation of the recovery of S → Fe(III) charge transfer after exposure to molecular oxygen is the formation of Fe(II). In agreement with this interpretation is the use of Mӧssbauer spectroscopy to evidence the formation of Fe(II) during the anaerobic disproportionation of ADO-Fe(III) and thiols to generate ADO-Fe(II) and disulfide products [18]. Catalytic turnover of model complexes under aerobic conditions interferes with kinetic measurements. Accordingly, we elected to perform all subsequent reactions and kinetics experiments under an anaerobic atmosphere to avoid aerobic oxidation of Fe(II) to Fe(III).

After confirming **1(aq)** generates transient model complexes similar to previous reports using non-chelated Fe(III), we turned to selecting a suitable buffer for kinetics measurements. Previous reports of Fe(III)–thiolate reaction kinetics used acidic or alkaline conditions to solubilize non-chelated Fe(III) ions [27–30]. It is well known that Fe(III) near pH ~ 7 requires supporting ligands to maintain solubility. Indeed, dissolving **1** in aqueous solution (in low mM range) near neutral pH produces a clear, yellow solution. These qualitative observations suggested the use of tacn-chelated Fe(III) permits the study of Fe(III)–thiolate reactivity near neutral pH.

### Optimizing a buffer system for kinetics experiments

Motivated by the hydrolytic stability of **1(aq)** near neutral pH, we optimized buffer conditions for kinetics experiments. Early attempts with Tris–HCl (pH 8.0) revealed a limitation with using cysteine methyl ester for kinetics measurements. Near pH 8 cysteine methyl ester converts to cysteine by ester hydrolysis which appears as a gradual blue-shifting of the absorbance maximum during model complex decay (Figure S1). Given the propensity of cysteine methyl ester to undergo hydrolysis, we were unable to use this ligand for accurate disproportionation measurements. We next turned to a sulfonate-based buffer, BES, due to its suitable buffer range (p*K*_a_ = 7.1) and relatively weakly coordinating sulfonate group. Monitoring S → Fe(III) charge transfer absorptions as a function of time in BES buffer at pH 7.5 demonstrates the timescale of complex formation is faster than our fastest measurement timescale (~ 5 s) for *N*-acetylcysteine, mercaptopropionate, cysteamine, and cysteine (Figure S2). Complex formation between **1(aq)** and penicillamine is observable as evidenced by increasing absorbance at 568 nm up to ~ 450 s. After this time period the complex exhibited second-order decay from 500–4000 s. Formation of complexes appears pH dependent as evidenced in two experiments performed in BES at pH 7.1 (Figure S3). The first involves cysteine which at pH 7.5 forms a complex faster than our measurement timescale, but complexation is observed to a small extent as pH 7.1. The second experiment involves penicillamine which appears to exhibit a modestly lengthened lag time before absorbance values increased, but the time interval before transitioning to second-order decay is similar at both pH values. These experiments suggest that complex formation is pH dependent as would be expected for generating a thiolate-bound Fe(III) center from a thiol. This further suggests that the rates of complex formation are dependent on thiol p*K*_a_. However, under our experimental conditions complex formation is either too rapid for observation or only somewhat observable alongside simultaneous disproportionation. Considering the focus of the study is disproportionation, it is worth emphasizing that during second-order decay there is no observable pH dependence, at least at the pH values examined here, which is consistent with a proton-independent disproportionation reaction. Overall, these observations suggest time intervals in BES at pH 7.5 in which reciprocal absorbance as a function of time exhibit high linearity (R^2^ ≥ 0.99) are appropriate for calculating disproportionation rate constants.

### Ligand-dependent Fe(III)–thiolate disproportionation kinetics

**Fig. 3:**
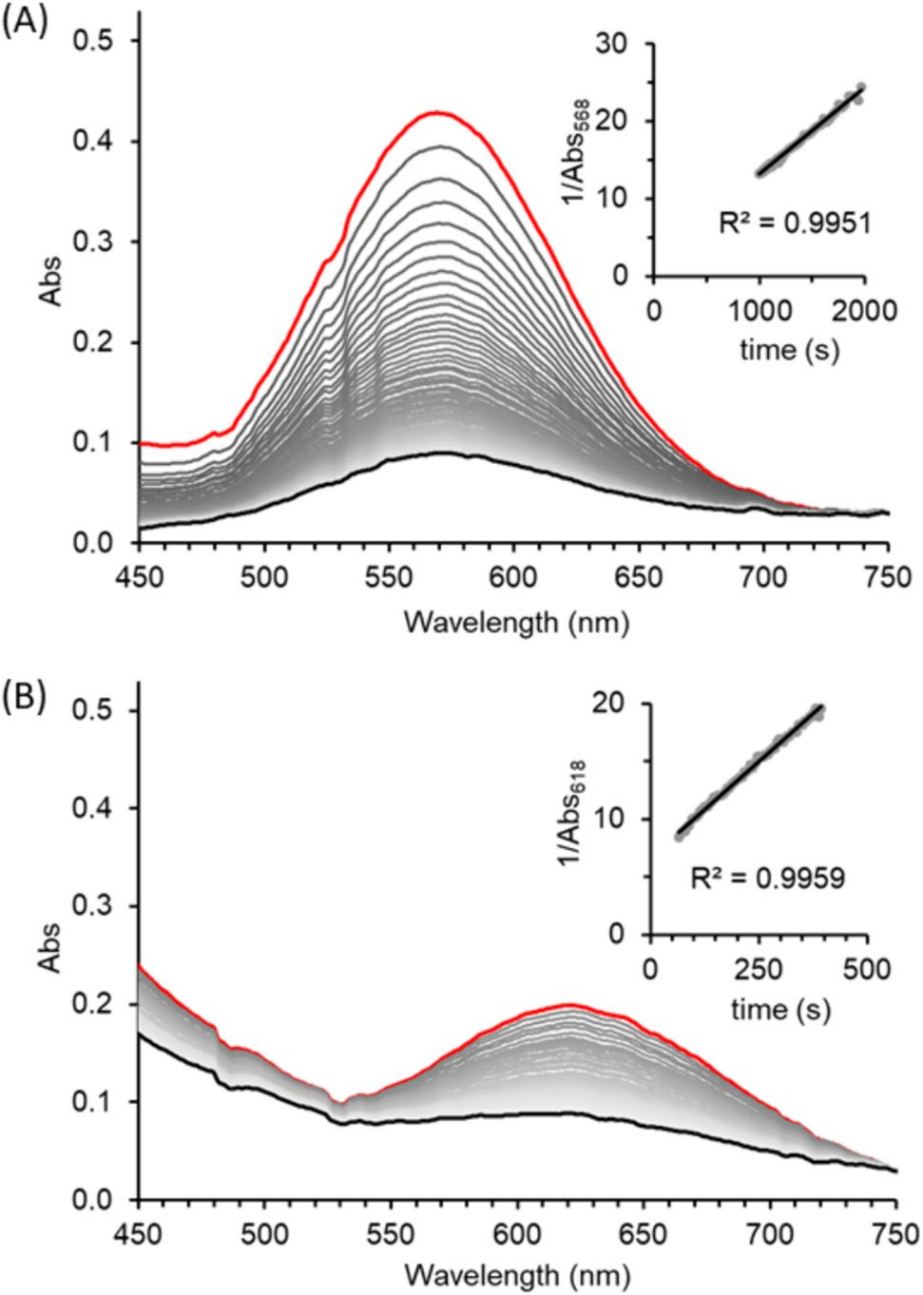
Representative UV–Vis spectra of **A** **1(aq)** + cysteine and **B** **1(aq)** + cysteamine with (inset) corresponding reciprocal absorbance values plotted as a function of time. Reactions were performed anaerobically and contained **1(aq)** (0.500 mM) and ligand (5.00 mM) in BES buffer (pH 7.5) at 37 °C

To measure the effect of thiol ligand on Fe(III)–thiolate disproportionation rates, we mixed **1(aq)** with thiol ligands (10 equivalents thiol) in pH 7.5 BES buffer at 37 °C under an anaerobic atmosphere and measured UV–Vis absorbance as a function of time. Disproportionation rates were calculated by measuring the linear regression slope of reciprocal absorbance maxima as a function of time. Reactions performed under these conditions generated disulfide products (see Figures S4 and S5 for representative data) and highly linear second-order rate plots exemplified by **1(aq)** + cysteine and **1(aq)** + cysteamine (Fig. 3). It is worth emphasizing that within these anaerobic reactions the only oxidant capable of generating disulfides from thiol/thiolates is Fe(III). This anaerobic reactivity and logic was also used in the discovery and characterization of ADO-Fe(III) disproportionation chemistry [18].

**Fig. 4:**
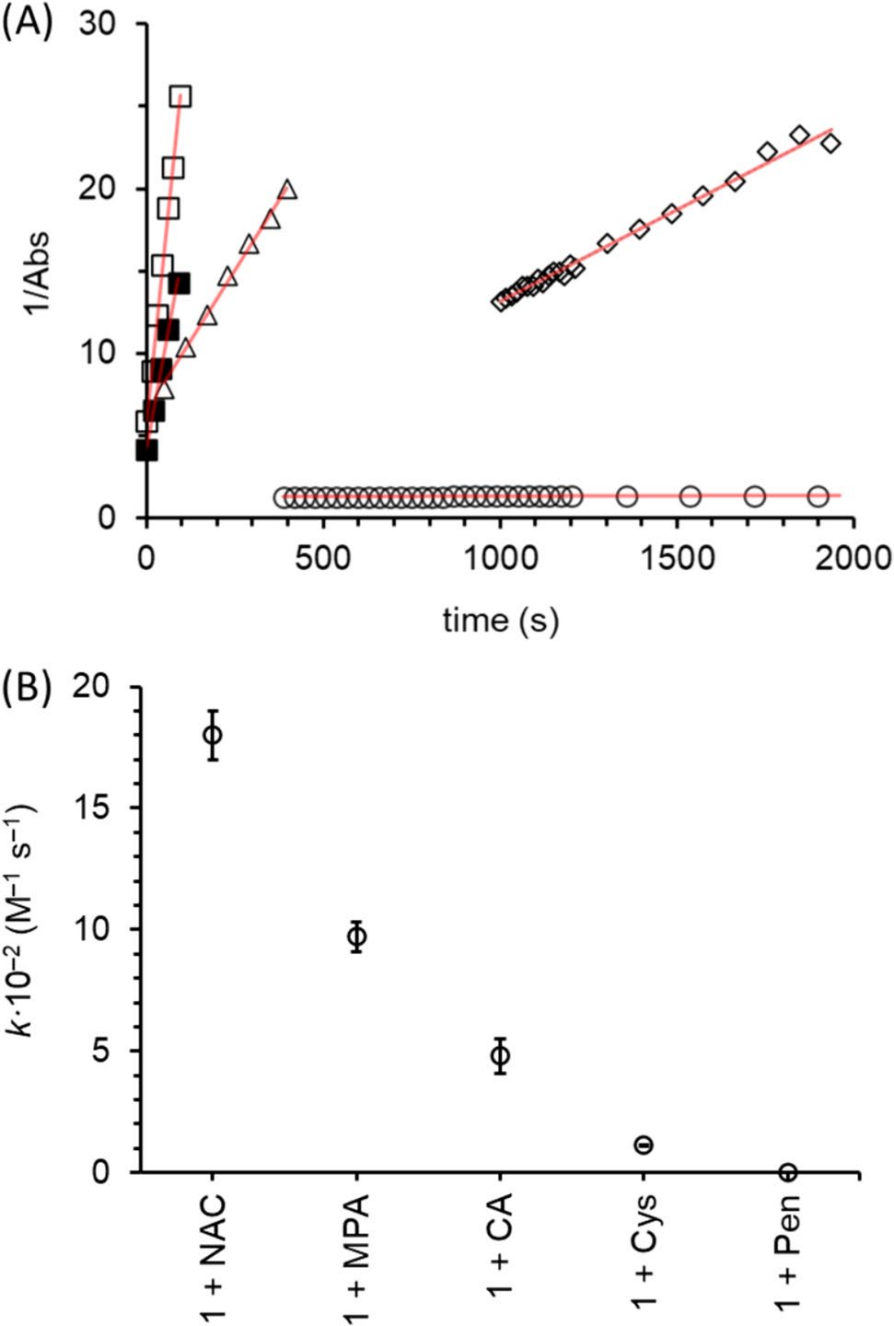
**A** Reciprocal absorbance as a function of time for **1(aq)** with *N*-acetylcysteine (open square), mercaptopropionate (filled square), cysteamine (open triangle), cysteine (open diamond), and penicillamine (open circle). Red lines represent linear regression fitting. **B** Second-order disproportionation rate constants as a function of reaction **1(aq)** with *N*-acetylcysteine (NAC), mercaptopropionate (MPA), cysteamine (CA), cysteine (Cys), and penicillamine (Pen). Error bars represent standard error of the mean (*n* = 3). Reactions were performed anaerobically and contained **1(aq)** (0.500 mM) and substrate (5.00 mM) in BES buffer (pH 7.5) at 37 °C

To evaluate trends in Fe(III)–thiolate disproportionation rates, we compared second-order rate plots and their corresponding rate constants (Fig. 4). Second-order rate plots illustrate Fe(III)–thiolate model complexes with relatively high and low reactivity. The highly reactive model complexes formed from **1(aq)** combined with *N*-acetylcysteine, mercaptopropionate, and cysteamine. Within the highly reactive series, *N*-acetylcysteine and mercaptopropionate produced the most reactive model complexes with disproportionation rate constants of 18 ± 1 × 10^–2^ M^–1^ s^–1^ and 9.7 ± 0.6 × 10^–2^ M^–1^ s^–1^, respectively, while the cysteamine-model complex decayed with a rate constant of 4.8 ± 0.7 × 10^–2^ M^–1^ s^–1^. Model complexes with relatively low reactivity were formed from cysteine and penicillamine which decayed with rate constants of 1.137 ± 0.003 × 10^–2^ M^–1^ s^–1^ and 0.009 ± 0.001 × 10^–2^ M^–1^ s^–1^, respectively.

To rationalize Fe(III)–thiolate disproportionation rates as a function of thiol ligand, it is helpful to consider an observation using *N*-acetylcysteine methyl ester. No S → Fe(III) charge-transfer absorption is observed between **1(aq)** and *N*-acetylcysteine methyl ester, suggesting this ligand interacts too weakly to establish an Fe(III)–thiolate bond. While interactions between amide or ester functional groups of *N*-acetylcysteine methyl ester and the Fe(III) center in **1(aq)** are possible, they appear insufficient for the ligand to establish an observable Fe(III)–thiolate interaction. Interestingly, while no Fe(III)–thiolate model complex is observed with *N*-acetylcysteine methyl ester, exchanging the methyl ester for a carboxylate (i.e., *N*-acetylcysteine) or exchanging the amide for an amine (i.e., cysteine methyl ester) both result in ligands capable of generating Fe(III)–thiolate model complexes. It appears that of the thiol ligands explored in this study, so long as a thiol is present with a carboxylate, amine, or both, Fe(III)–thiolate model complexes are generated. Collectively, these observations suggest that, while the exact binding modes are unknown, multidentate binding (i.e., chelate effect) stabilizes the formation of Fe(III)–thiolate model complexes which then undergo disproportionation. Given the relative stability of some Fe(III)–thiolate model complexes, we performed EPR spectroscopy to gain insight into their electronic structures.

### EPR spectra of Fe(III)–thiolate model complexes

**Fig. 5:**
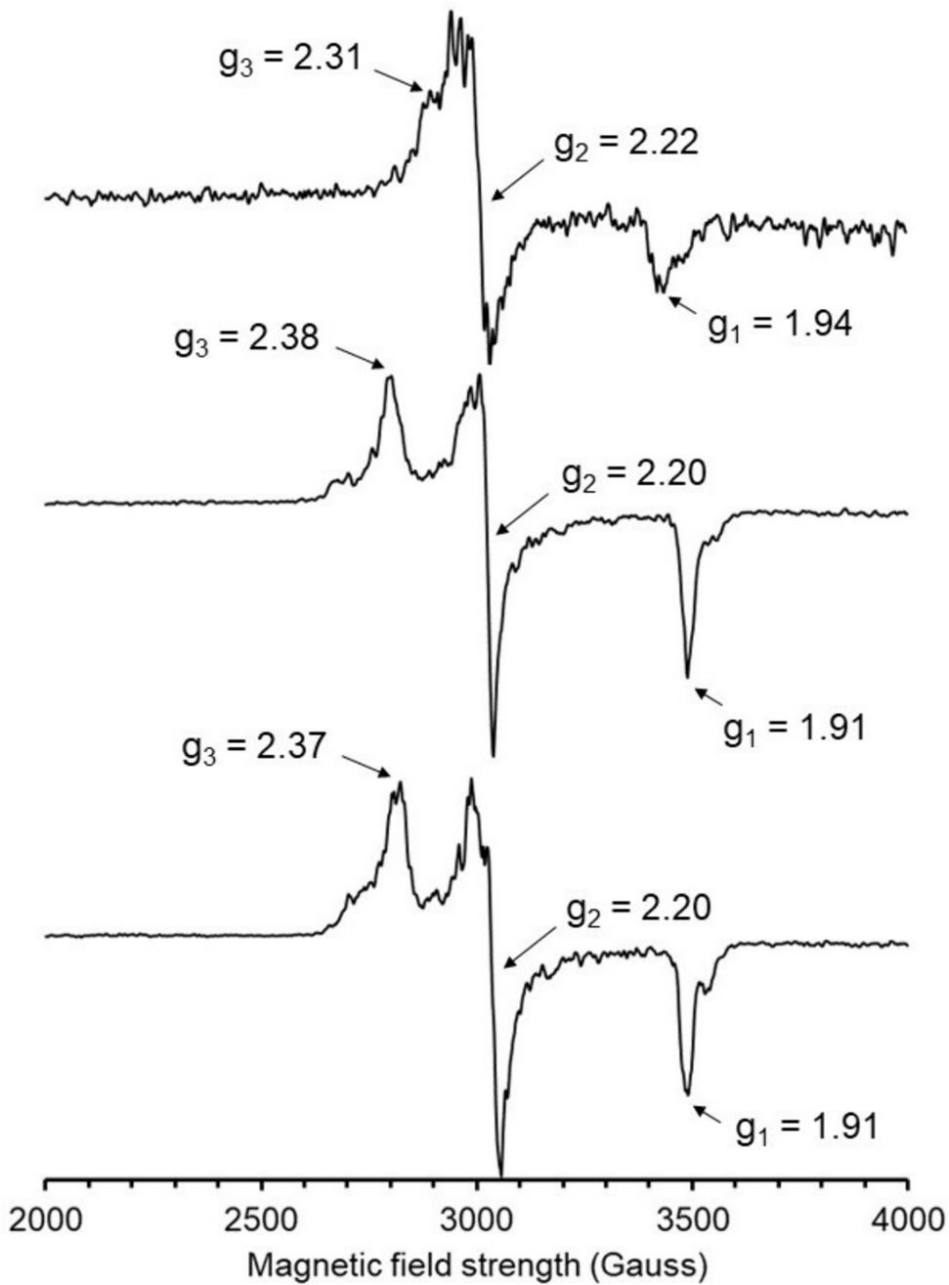
EPR spectra of model complexes generated with **1(aq)** combined with cysteamine (top), cysteine (middle), and penicillamine (bottom). Acquisition parameters include a sample temperature of 77 K, microwave frequency of 9.3454 GHz, modulation amplitude of 10 G, microwave power of 2 mW, and 64 scans

Paramagnetic spectra were only observed for Fe(III)–thiolate model complexes generated with cysteamine, cysteine, and penicillamine (Figs. 5 and S6), which are the most stable model complexes based on disproportionation rates. Signal intensity was highest for the most stable model complexes containing cysteine and penicillamine, and lowest for the model complex containing cysteamine. Model complexes containing cysteine and penicillamine exhibit similar rhombic EPR spectra with *g*_1_ = 1.91, *g*_2_ = 2.20, and *g*_3_ = 2.38 and *g*_1_ = 1.91, *g*_2_ = 2.20, and *g*_3_ = 2.37, respectively. Compared with the cysteine and penicillamine model complexes, the model complex containing cysteamine exhibits a less rhombic, more axial EPR spectrum with *g*_1_ = 1.94, *g*_2_ = 2.22, and *g*_3_ = 2.31. It is worth discussing some features of the EPR spectra reported here with respect to structural heterogeneity and relatively low signal intensity. First, the presence of shoulders in all three spectra suggest the presence of a second low-spin Fe(III) site in each sample. This suggests non-uniform binding modes of penicillamine, cysteine, and cysteamine at the Fe(III) center in **1(aq)**. Indeed, our system is incapable of replicating secondary coordination spheres of thiol dioxygenase active sites which contain highly conserved residues to orient substrates for binding. With respect to signal intensity, the spectra reported here have lower intensity and therefore higher noise than would be expected for low-spin Fe(III) at 10 mM concentration. The contributing factors are likely a combination of the reactivity of the model complexes, especially with respect to the sample containing cysteamine, and the EPR-silent nature of a reaction product containing low-spin Fe(II). In the case of cysteamine, the disproportionation is rapid and for this reason it is highly plausible that the effective concentration of Fe(III) in the sample is significantly lower than the total Fe concentration. For all three samples there is the possibility of aggregation of complexes leading to antiferromagnetic coupling, which again would account for an effective concentration of mononuclear low-spin Fe(III) lower than the total Fe concentration. For these reasons simulations are likely of limited value but at a minimum all three Fe(III)–thiolate model complexes detected by EPR are consistent with low-spin Fe(III) centers and are also consistent with EPR spectra reported for ADO-Fe(III). For example, ADO-Fe(III) in the presence of cysteamine and cyanide (a strong-field ligand used as a surrogate for superoxide) exhibits a predominantly low-spin Fe(III) signal with *g*_1_ = 1.96, *g*_2_ = 2.20, and *g*_3_ = 2.30 [8]. It should be noted that low-spin Fe(III) is observed only when ADO-Fe(III) is exposed to a thiol substrate and strong-field ligand, but otherwise exhibits high-spin Fe(III) signals. Interestingly, a minor g = 4.3 signal is observed in a subset of our Fe(III)–thiolate EPR spectra suggesting the minor presence of high-spin Fe(III). Despite the spin-state transition from high to low spin observed in ADO-Fe(III), it is highly common to observe a relatively small amount of high-spin signal around g = 4.3. To gain deeper insight our measured Fe(III)–thiolate disproportionation rates, we turned to cyclic voltammetry to characterize electrochemical properties of the Fe(III)–thiolate model complexes.

### Cathodic potentials correlate with disproportionation rates

**Fig. 6:**
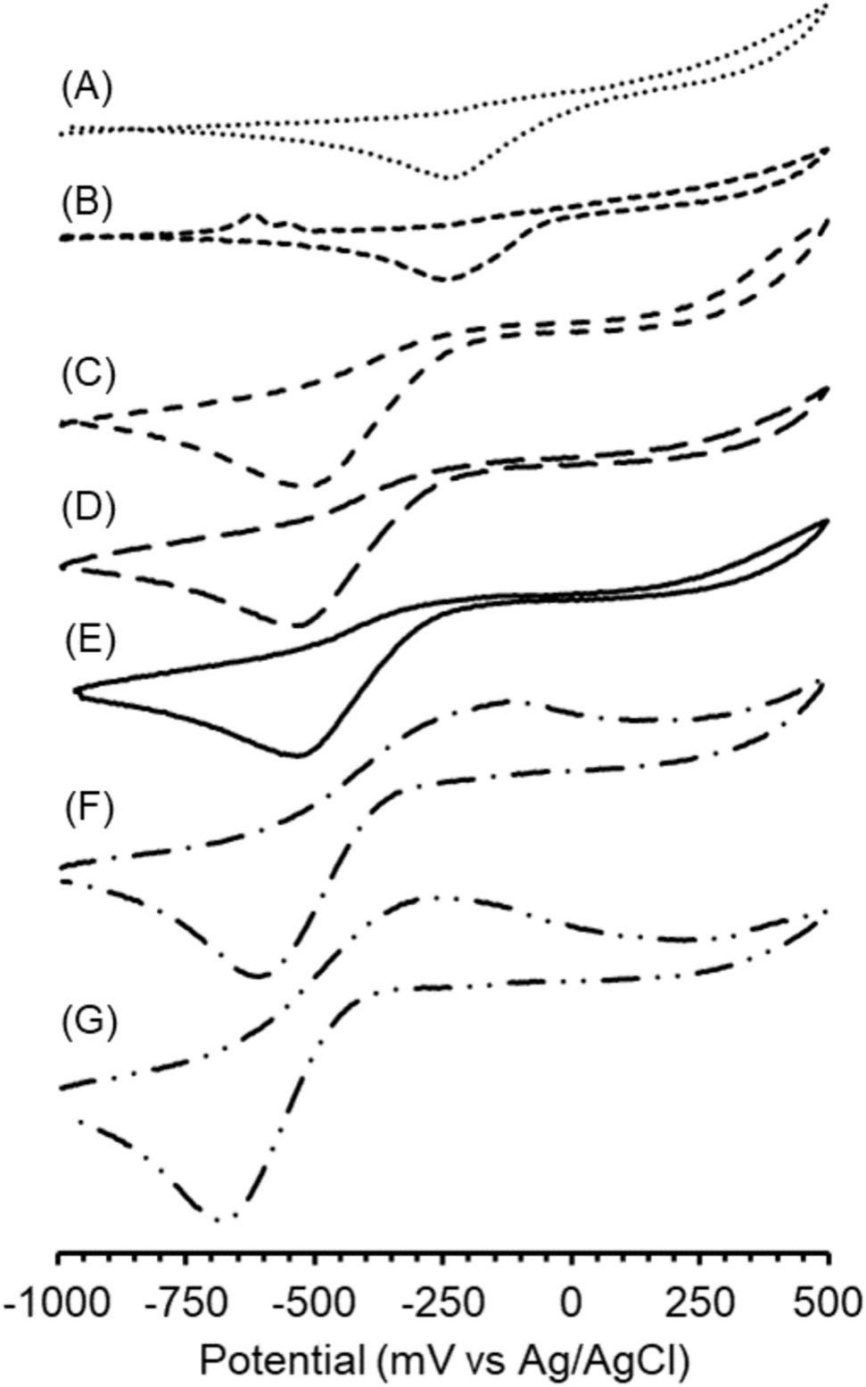
Cyclic voltammograms of samples containing **A** **1(aq)** + *N*-acetylcysteine (. . .), (B) **1(aq)** + mercaptopropionate (- - -), **C** **1(aq)** + cysteamine (– – –), **D** **1(aq)** + *N*-acetylcysteine methyl ester (— — —), **E** **1(aq)** (—), **F** **1(aq)** + cysteine (— ⋅ —), and (G) **1(aq)** + penicillamine (— ⋅ ⋅ —). All samples are in BES buffer (20 mM BES, 138 mM NaCl, pH 7.5), were prepared and measured under anaerobic conditions (N_2_ atmosphere, 2–4% H_2_, < 1 ppm O_2_), and were acquired with a 750 mV/s scan rate

To measure electrochemical properties of Fe(III)–thiolate model complexes, we turned to cyclic voltammetry. We hypothesized that the most reactive model complexes contain the most oxidizing Fe(III) centers and would therefore exhibit the most positive cathodic potentials. Cyclic voltammograms of **1(aq)** alone or in the presence of thiol ligands generally exhibit irreversible voltammograms, but model complexes generated with cysteine and penicillamine exhibit quasireversible characteristics (Fig. 6). Given the irreversible or quasireversible nature of the cyclic voltammograms, we elected to compare cathodic peak potentials.

Comparing cathodic peak potentials with disproportionation rate constants reveals a clear trend where more positive cathodic peak potentials correspond to more reactive Fe(III)–thiolate model complexes (Table 1). The correlation between cathodic peak potential and reactivity strongly suggests the cathodic current arises from the reduction of the Fe(III) center. This interpretation can also explain the irreversibility of the cyclic voltammograms because electrochemical reduction of Fe(III) to Fe(II) coincides with a shift from a hard to relatively soft Lewis acid which might lead to a structural change, such as the dissociation of a ligand, from the model complex. Interestingly, the most reactive model complexes generated with *N*-acetylcysteine and mercaptopropionate exhibit indistinguishable cathodic peak potentials but have distinguishable disproportionation rate constants. The most stable model complexes generated with cysteine and penicillamine exhibit a similar phenomenon with relatively similar cathodic peak potentials but markedly different disproportionation rates. These observations suggest that Fe(III)–thiolate disproportionation rate is sensitive not only to electronic structure of the Fe(III) center but also to steric effects of the thiol ligand itself. Given the sensitivity to steric effects of the thiol ligand, these results agree with bimolecular disproportionation.

**Table 1:** Disproportionation rate constants and cathodic peak potentials for Fe(III)–thiolate model complexes and corresponding controls

Sample	S → Fe(III) λ_max_ (nm)	Disproportionation rate constant (× 10^–2^ M^–1^ s^–1^)	Cathodic peak potential (mV vs Ag/AgCl)
**1(aq)** + *N*-acetylcysteine	568	18 ± 1	− 202 ± 1
**1(aq)** + mercaptopropionate	568	9.7 ± 0.6	− 202 ± 4
**1(aq)** + cysteamine	618	4.8 ± 0.7	− 516 ± 3
**1(aq)** + cysteine methyl ester	618	N/A	N/A
**1(aq)**	N/A	N/A	− 531 ± 3
**1(aq)** + *N*-acetylcysteine methyl ester	N/A	N/A	− 534 ± 1
**1(aq)** + cysteine	568	1.137 ± 0.003	− 609 ± 1
**1(aq)** + penicillamine	568	0.009 ± 0.001	− 682 ± 5

Values are reported as the mean ± standard error (*n* = 3) for independently prepared samples

### Isolation of a stable Fe(III)–thiolate model complex

Motivated by the stability of the model complex generated with **1(aq)** and cysteine, we attempted to isolate it from aqueous solution. The cysteine model complex appeared to precipitate in the presence of tetraphenylborate. However, the light blue precipitate immediately decomposed to yellow-green products in polar organic solvents, such as tetrahydrofuran and acetonitrile, making purification and characterization challenging. Given the structural similarity of cysteine and penicillamine, the highly similar EPR spectra of both cysteine- and penicillamine-containing model complexes, and the apparent stability of the penicillamine model complex, we reasoned that isolation and characterization of the penicillamine model complex would be feasible and informative. Isolation of the penicillamine was achieved through precipitation with sodium tetraphenylborate to yield a light blue powder. The light blue powder was insoluble in water but soluble and moderately stable in tetrahydrofuran and acetonitrile yielding deep purple solutions. This observation suggested crude **2** was isolated. Precipitation of **2** from tetrahydrofuran with diethyl ether yields a dark purple powder that, based on elemental analysis, is consistent with the formula [Fe(tacn)(pen)]BPh_4_·H_2_O. Having isolated penicillamine model complex **2**, we next turned to characterization of its coordination environment and electronic structure.

**Fig. 7:**
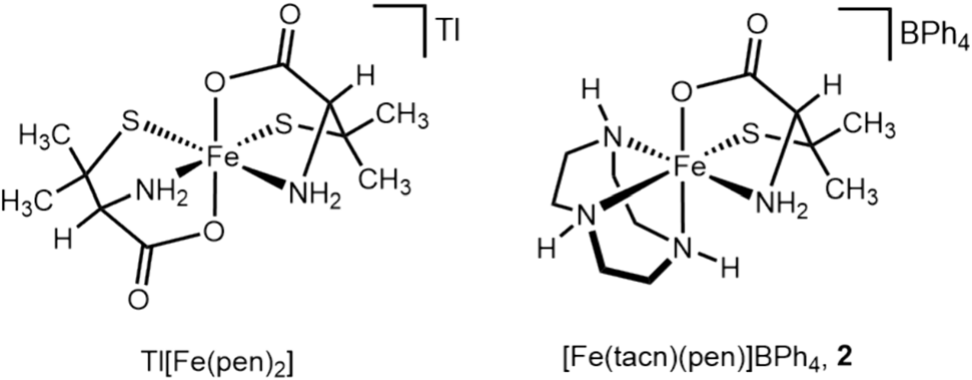
Structure of Tl[Fe(pen)_2_], a crystallographically characterized complex containing two penicillamine ligands each bound a tridentate mode [44], and the proposed structure of complex **2**

To characterize the coordination environment of **2**, we performed infrared spectroscopy. The FT-IR spectrum of **2** contains notable features at 3433, 3242, and 1646 cm^–1^ corresponding to hydrate O–H, tacn N–H, and penicillamine C=O frequencies, respectively (Figure S7). These assignments agree well with those previously reported for the crystallographically characterized Fe(III)-penicillamine complex Tl[Fe(pen)_2_] (Fig. 7) [44]. In Tl[Fe(pen)_2_] the penicillamine N–H and C=O frequencies are assigned at 3270 and 1640 cm^–1^, respectively. We assign the stretch at 3242 cm^–1^ as tacn N–H but do observe a shoulder at ~ 3275 cm^–1^ which might arise from the penicillamine N–H stretch. Collectively, the FT-IR frequencies described here support the assignment of **2** containing penicillamine bound in a tridentate mode (Fig. 6). Moreover, given the similar structures of penicillamine and cysteine, it is likely that the cysteine model complex binds in a similar manner. When combined, our measured disproportionation rates, cathodic peak potentials, and characterization of the penicillamine-containing model complex suggest tridentate binding decreases Fe(III)–thiolate disproportionation rate primarily through electronic effects at the metal center. After characterizing vibrational features, we next sought to characterize the electronic structure of **2**.

To characterize the electronic structure of **2**, we acquired UV–Vis, EPR, and NMR spectra (Figures S8–S10) in addition to performing solid state and solution phase magnetic susceptibility measurements. In acetonitrile complex **2** exhibits a S → Fe(III) charge transfer absorption at 585 nm representing a 17 nm red shift relative to the violet colored model complexes generated in aqueous solution. The EPR spectrum of complex **2** is consistent with a low-spin Fe(III) center also observed for model complexes generated from **1(aq)** and cysteamine, cysteine, and penicillamine. Solid-state magnetic susceptibility measurements were performed using **2** in its powder form within a magnetic susceptibility balance and solution phase measurements were performed using the Evans NMR method. The effective magnetic moment calculated for solid and solution samples of **2** were 1.67 and 1.68 *µ*_B_, respectively, indicating that **2** contains a low spin (*S* = ½) Fe(III) center. In agreement with this electronic structure determination is the low-spin EPR spectrum of the model complex generated from **1(aq)** and penicillamine, the EPR spectrum of **2**, and the NMR spectrum of **2** (Figure S9) featuring broad proton resonances spanning –30 to 30 ppm consistent with other low-spin Fe(III) complexes [45]. Collectively, these results indicate **2** contains penicillamine bound to a low-spin Fe(III) center.

### Results within the context of thiol dioxygenases

Our results must be carefully interpreted within the context of thiol dioxygenase disproportionation chemistry because our model system does not replicate secondary coordination sphere effects of thiol dioxygenase active sites. Moreover, ADO likely binds thiol substrates in a monodentate fashion [7, 18], which is not replicated by our study. However, there are several similarities between our results and characteristics of disproportionation chemistry exhibited by ADO. For example, the disproportionation timescale of ADO-Fe(III) with cysteamine and the N-terminus of RGS5 is on the order of minutes, where ADO-Fe(III) is reduced to ADO-Fe(II) to some extent after 1 min but nears completion after 10 min. [18] Our observations show a similar disproportionation timescale for the model complex generated from **1(aq)** + cysteamine where the S → Fe(III) charge transfer absorption decreases by ~ 50% after 1 min and ~ 90% after 8 min. In addition to reaction timescale, our measured disproportionation rates and cathodic peak potentials suggest that, in the absence of active- site secondary structure, Fe(III)–thiolate disproportionation is fastest when a thiol and carboxylate can bind the metal center, is slower when a thiol and amine can bind the metal center, and is slowest when a thiol, carboxylate, and amine can all access the metal center. From these observations it appears that Fe(III)–thiolate disproportionation is faster with thiol ligands that bind with lower denticity. Interestingly, while ADO-Fe(III) undergoes disproportionation chemistry with its native substrates, cysteine bound to CDO-Fe(III) forms a stable complex [46, 47]. Given that native substrates bind to ADO-Fe(III) and CDO-Fe(III) in a monodentate and bidentate mode, respectively, our results suggest this difference in disproportionation reactivity might be due to lower substrate denticity at the ADO-Fe(III) active site.

## Conclusion

We examined the effect of a series of thiol ligands on Fe(III)–thiolate disproportionation rates. Measuring disproportionation rates is relevant to the chemistry of ADO because of its unique substrate selectivity and recently discovered disproportionation chemistry. We used **1** as a starting point because it contains a 3N facial coordination environment and exhibits moderate hydrolytic stability as **1(aq)** near neutral pH. Previous studies on Fe(III)–thiolate disproportionation reactions used non-chelated Fe(III) necessitating acidic or alkaline solutions to maintain Fe(III) solubility. Thiol dioxygenases bind substrates in a physiologically relevant pH range, so results in acidic and alkaline solution are limited within the context of modeling ADO disproportionation chemistry. Based on results from our kinetic and electrochemical studies, it appears that Fe(III)–thiolate disproportionation rates and cathodic peak potentials depend on ligand functional groups where thiol/carboxylate > thiol/amine > thiol/carboxylate/amine. We detected the three most stable model complexes by EPR spectroscopy demonstrating low-spin Fe(III) centers. Furthermore, we observe disproportionation reactivity on the same timescale reported for ADO disproportionation activity. To support the assignment of cysteine and penicillamine binding in a tridentate mode, we isolated the penicillamine-containing model complex **2** as a tetraphenylborate salt. Complex **2** was characterized by elemental analysis, ^1^H NMR, FT-IR, UV–Vis, and EPR spectroscopies, and magnetic susceptibility measurements which collectively suggest penicillamine is bound tridentate to a low-spin Fe(III) center.

Collectively, our experiments bridge the gap between seminal work on Fe(III)–thiolate disproportionation kinetics and modern mimetic chemistry using supporting ligands. These results will be useful in rationalizing disproportionation kinetics, cathodic peak potentials, and electronic structure of ADO-Fe(III) and other non-heme Fe metalloenzymes exhibiting Fe(III)–thiolate disproportionation chemistry.

### Supplementary Information

Below is the link to the electronic supplementary material.

Supplementary file1 (PDF 687 KB)

## Data Availability

The authors declare that the data supporting the findings of this study are available within the paper and its Supplementary Information files. Should any raw data files be needed they are available from the corresponding author upon reasonable request.
