# Supplementary material for: Spectroscopic, electrochemical, and kinetic trends in Fe(III)–thiolate disproportionation near physiologic pH
Source: J Biol Inorg Chem. 2024 May 9;29(3):291–301. doi: 10.1007/s00775-024-02051-3 (PMC11111527; doi:10.1007/s00775-024-02051-3)
Supplement: Supplementary file 1 — Supplementary file1 (PDF 687 KB) [file 775_2024_2051_MOESM1_ESM.pdf]

Supporting Information

Spectroscopic, Electrochemical, and Kinetic Trends in Fe(III)–Thiolate Disproportionation Near Physiological pH

Levi A. Ekanger,* Ruhi K. Shah, Matthew E. Porowski, Zach Ziolkowski, and Alana Calello

Contribution from the Department of Chemistry,
The College of New Jersey, Ewing, NJ 08628, United States

*E-mail: ekangerl@tcnj.edu

**Page**	**Contents**
S1	Table of Contents
S2	UV-vis spectra of **1(aq)** + cysteine methyl ester
S3	Representative absorbance vs time plots
S4	Absorbance vs time at pH 7.5 and 7.1
S4	NMR spectra of cysteine to cystine conversion
S5	Mass spectra demonstrating disulfide formation
S6	EPR spectra
S7	FT-IR spectrum of **2**
S7	UV-vis spectrum of **2**
S8	EPR spectrum of **2**
S8	NMR spectra of **2**

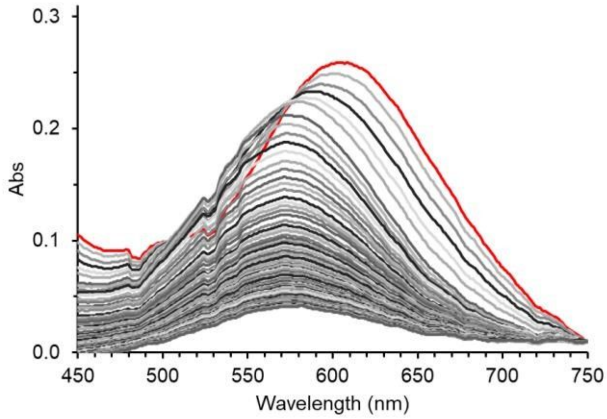

Figure S1. Representative UV-vis spectra of **1(aq)** + cysteine methyl ester (37 °C, BES pH 7.5) demonstrating a blue shift concomitant with decay of the S→Fe charge transfer absorption of the complex. The blue shift is consistent with hydrolysis of cysteine methyl ester generating cysteine.

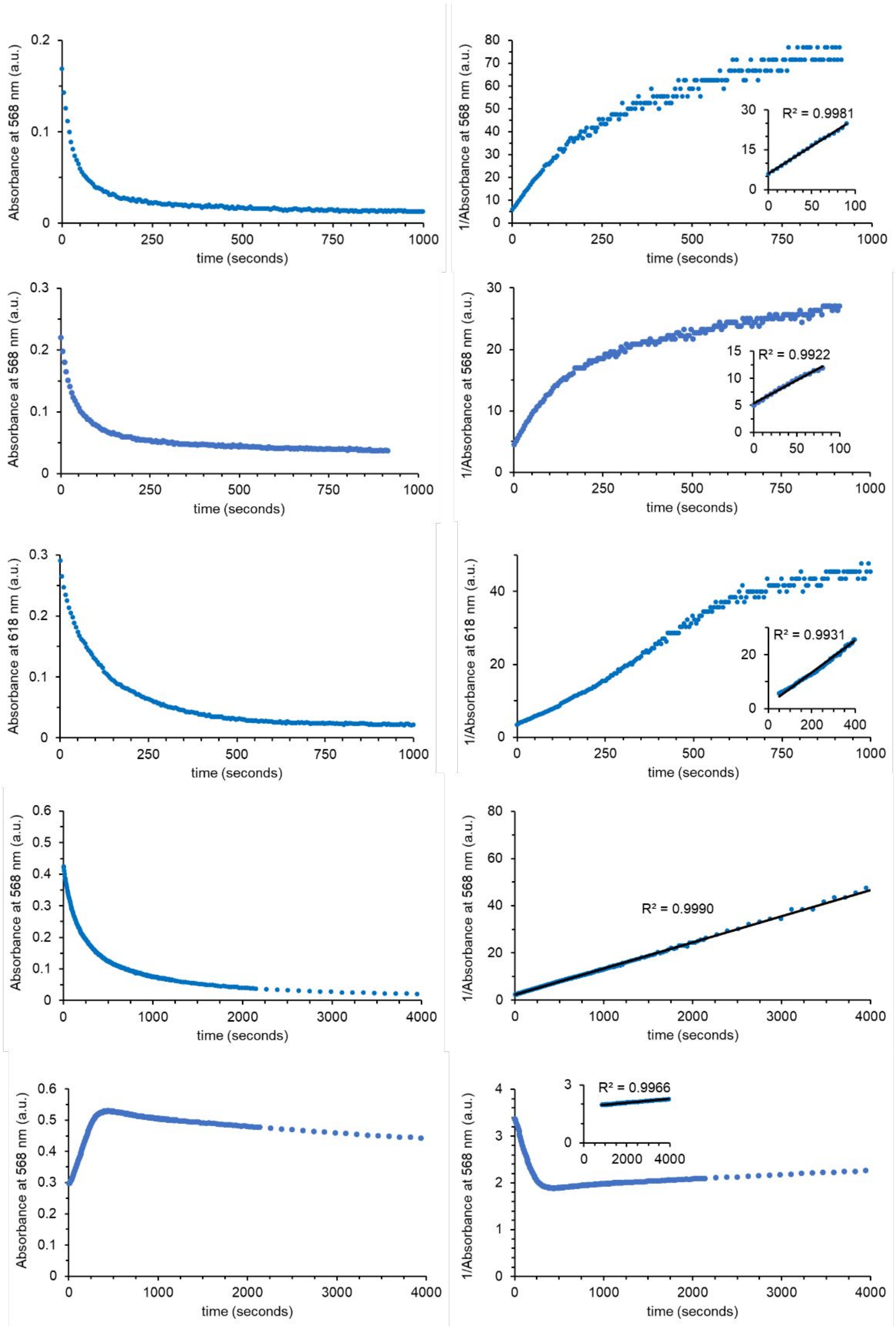

Figure S2. Representative absorbance and reciprocal absorbance as a function of time plots for **1(aq)** after mixing with (top to bottom) *N*-acetylcysteine, mercaptopropionate, cysteamine, cysteine, and penicillamine. Insets show time intervals with high linearity in second order decay except for the reaction with cysteine which exhibited highly linear second-order decay for the entire experiment duration.

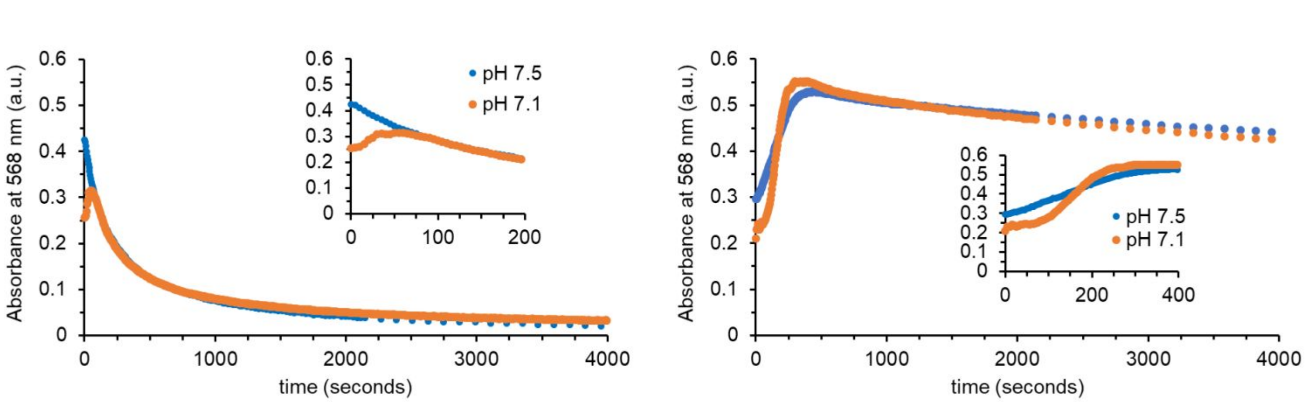

Figure S3. Representative absorbance as a function of time plots for **1(aq)** after mixing with cysteine (left) and penicillamine (right) at pH 7.5 and pH 7.1. A pH dependence is observed with respect to complex formation, but the decay by disproportionation appears insensitive to pH.

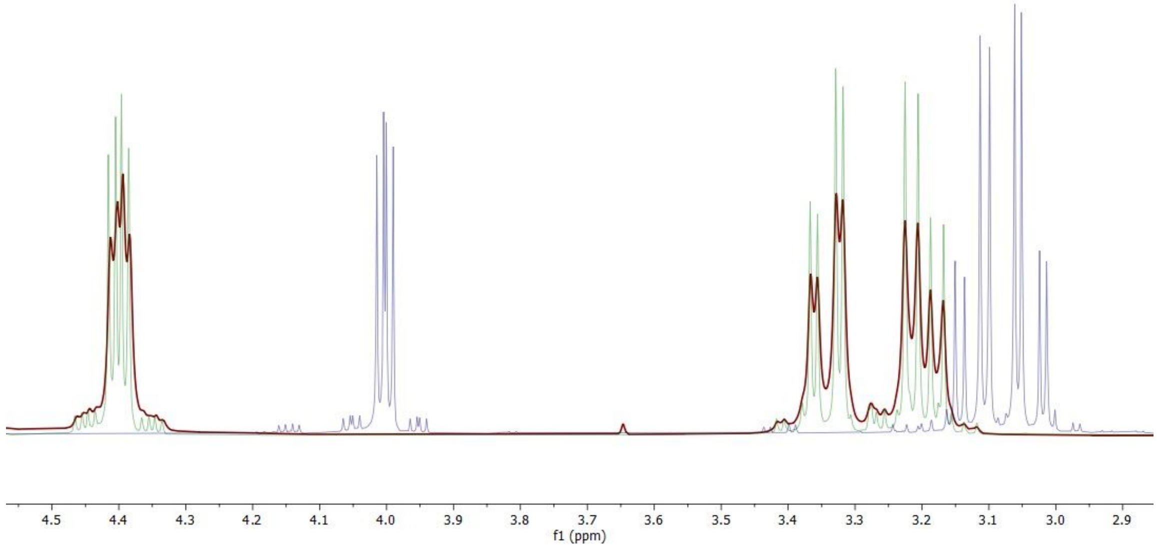

Figure S4. ^{1}H-NMR spectra (D_2O, 400 MHz) of cysteine (blue), cystine (green), and the reaction product of **1(aq)** + cysteine (dark red) isolated as a precipitate. Cystine and reaction product were dissolved through the dropwise addition of DCl (20% w/w solution in D_2O) until all solids dissolved (~1–3 drops).

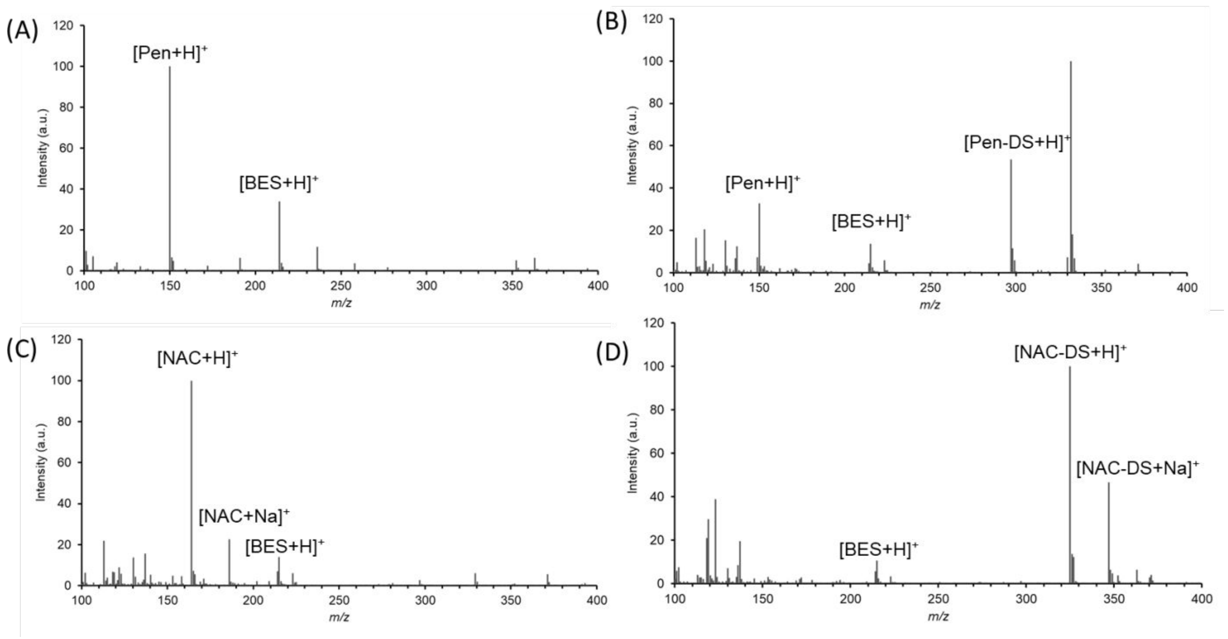

Figure S5. Representative mass spectra demonstrating thiol-to-sulfide conversion resulting from disproportionation. (A) Mass spectrum (elution time = 1.1 min) of penicillamine (Pen, M = 149 Da), (B) mass spectrum (elution time = 2.9 min) of **1(aq)** + penicillamine generating its disulfide (Pen-DS, M = 296 Da), (C) mass spectrum (elution time = 2.5 min) of *N*-acetylcysteine (NAC, M = 163 Da), and (D) mass spectrum (elution time = 5.7 min) of **1(aq)** + *N*-acetylcysteine generating its disulfide (NAC-DS, M = 324 Da). Reaction buffer *N*,*N*-bis(2-hydroxyehtyl)-2-aminoethanesulfonate (BES, M = 213 Da) is observed through all mass spectra at varying intensities.

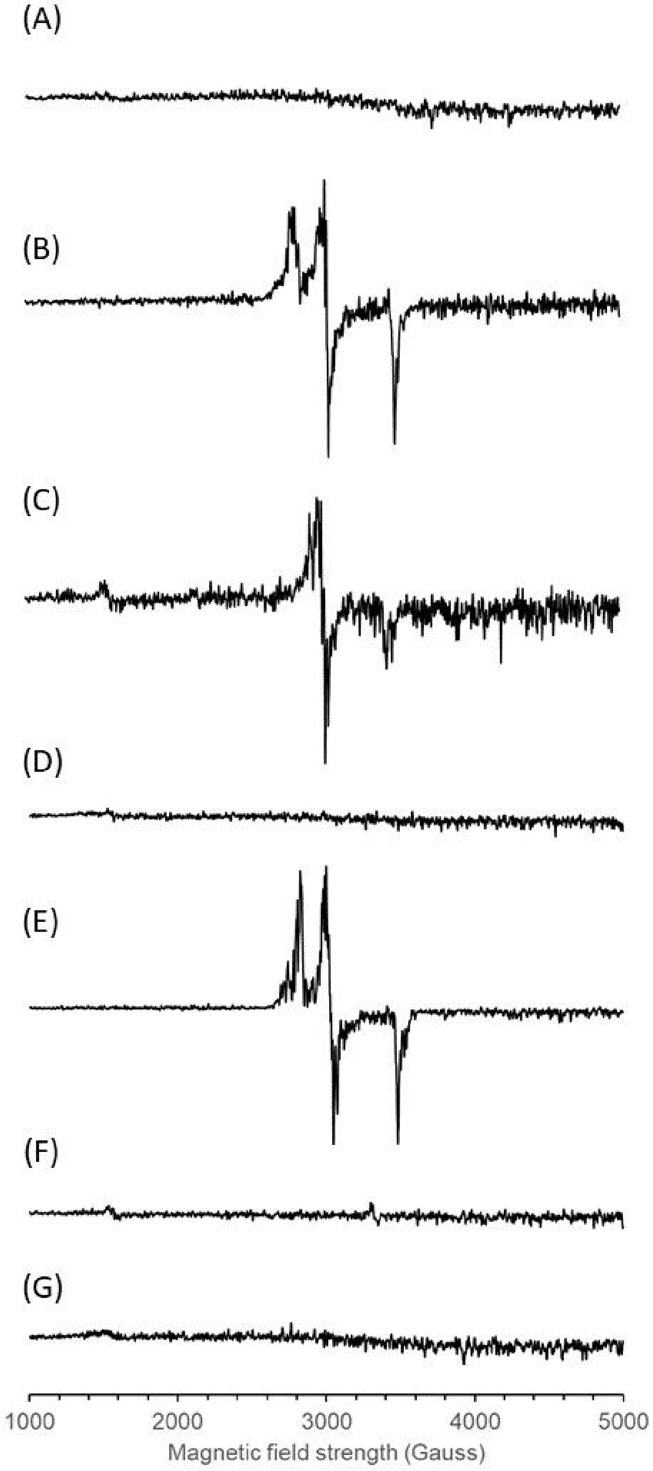

Figure S6. Wide-spectral-width EPR spectra of **1(aq)** alone (A) or in combination with cysteine (B), cysteamine (C), mercaptopropionate (D), penicillamine (E), *N*-acetylcysteine (F), and *N*-acetylcysteine methyl ester (G). Acquisition parameters include a sample temperature of 77 K, microwave frequency of 9.3454 GHz, modulation amplitude of 10 G, microwave power of 0.63 mW, and 10 scans.

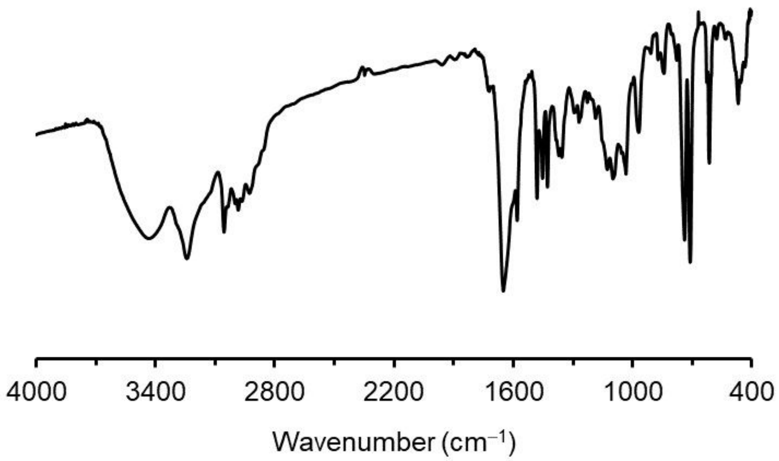

Figure S7. FT-IR spectrum of **2** in KBr pellet.

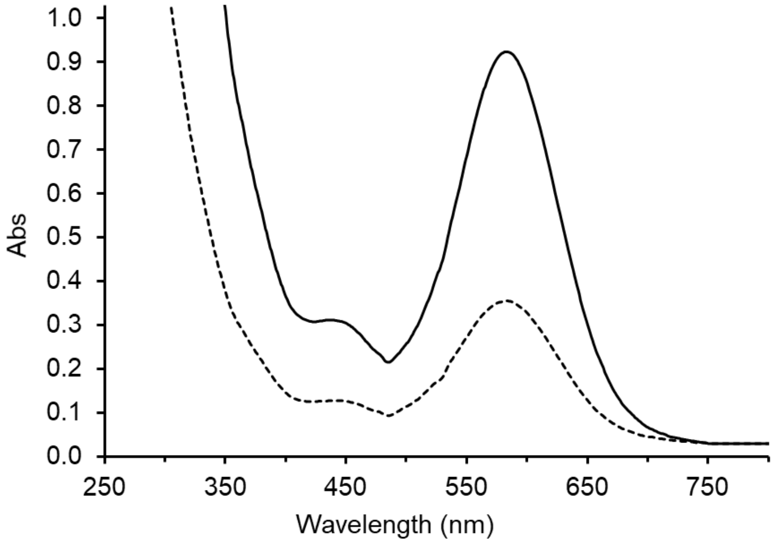

Figure S8. UV-vis spectra of **2** in acetonitrile at concentrations of 360 µM (solid line) and 120 µM (dashed line).

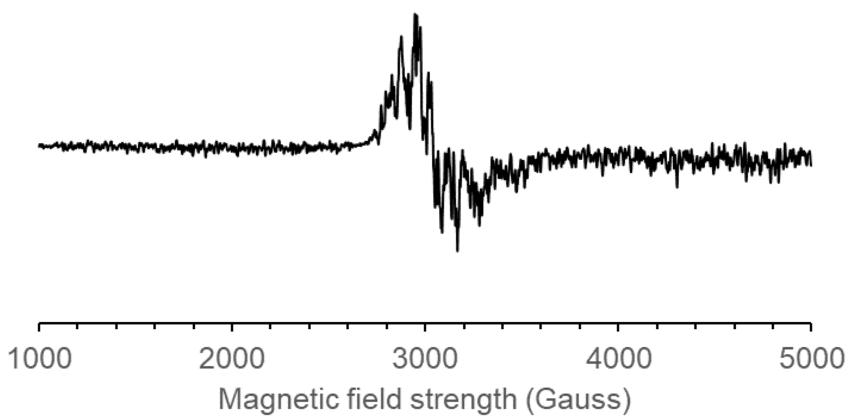

Figure S9. EPR spectrum of **2** (7 mM) in acetonitrile. Acquisition parameters include a sample temperature of 77 K, microwave frequency of 9.440 GHz, modulation amplitude of 10 G, microwave power of 0.63 mW, and 10 scans.

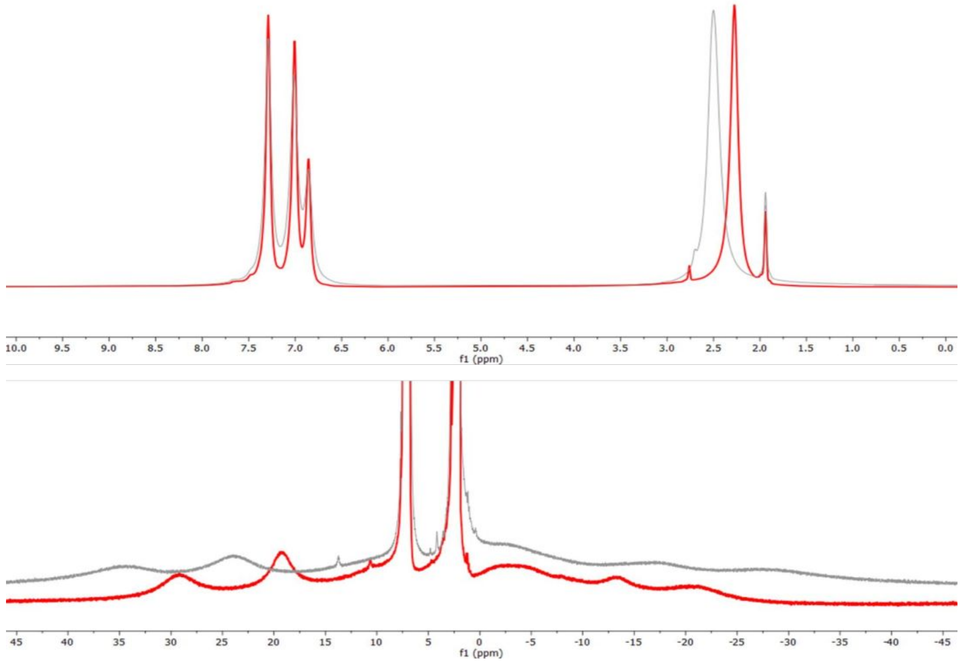

Figure S10. ^{1}H-NMR spectra (CD_3CN, 400 MHz) of **2** at 17 °C (red) and –20 °C (gray) scaled to the highest intensity signals in the diamagnetic region (top) and to low-intensity signals in the paramagnetic region (bottom). Broad, paramagnetic resonances exhibited relatively large (~5 ppm) shifts upon temperature change. The diamagnetic region contains proton resonances from tetraphenylborate counterion near 7.0 ppm (chemical shift unperturbed by temperature change and no observable coupling) and residual water near 2.2 ppm exhibiting a small shift (0.2–0.3 ppm) by temperature change.
